# Case report: Sudden cardiorespiratory collapse in a healthy male after coronavirus disease 2019 vaccination at a vaccination center

**DOI:** 10.3389/fcvm.2022.1014250

**Published:** 2022-09-29

**Authors:** Cze Ci Chan, Chia-Pin Lin, Chi-Jen Chang, Pao-Hsien Chu

**Affiliations:** Department of Cardiology, Linkou Medical Center, Chang Gung Memorial Hospital, Taoyuan City, Taiwan

**Keywords:** coronary artery disease, sudden collapse, COVID-19 ChAdOx1 nCoV-19 vaccine, COVID-19 vaccination adverse effect, cardiovascular adverse events, case report

## Abstract

Since 2020, new vaccines were developed to fight the coronavirus disease 2019 (COVID-19). Vaccination is important in preventing mortality and achieving herd immunity. However, due to vast vaccination, fatal adverse events could be seen. We report a case of a previously healthy, young male who had a cardiopulmonary arrest 2 min after receiving the Oxford- AstraZeneca (ChAdOx1 nCoV-19) COVID-19 vaccination. After targeted temperature management, a coronary angiogram was performed after neurological recovery and showed severe stenosis at the proximal left anterior descending artery. Stenting was done and he was discharge. No similar case of sudden cardiorespiratory collapse immediately after COVID-19 vaccination has been reported. Our patient did not have any effort-related angina or dyspnea on exertion before this event. The sudden cardiorespiratory collapse was probably related to underlying coronary artery disease, complicated with a vasovagal event. We stress the importance of coronary angiography in out of hospital cardiac arrest patients after neurological recovery. In the era of COVID-19 vaccination, even though fatal adverse events following immunization are rare, heightened awareness of severe side effects needing medical attention is very important.

## Introduction

Since 2020, the coronavirus disease 2019 (COVID-19) pandemic has placed a heavy burden on healthcare systems worldwide. The number of cases continues to increase daily, and several measures have been taken to combat this virus. New vaccines were developed with extraordinary speed to stop the virus and prevent more deaths. Taiwan has now approved four vaccines, Moderna, BioNTech, Oxford-AstraZeneca and Medigen (1). In July 2021, total number of people with at least one dose of COVID-19 vaccine in Taiwan and worldwide were 6.43 million (24.6%) and 2.15 billion (26.8%) respectively. Two hundred and forty thousand people are vaccinated daily then to cope with the rapidly spreading disease in Taiwan, with the majority being vaccinated with either Moderna or Oxford-AstraZeneca vaccine (2). Although the vaccines are beneficial in reducing COVID-19 severity, some rare complications have been reported (3). Here, we report a case of sudden collapse after vaccination at a vaccination center, with the aim of generating awareness among clinicians and other healthcare workers regarding rare but potentially fatal events.

## Case description

**FIGURE 1:**
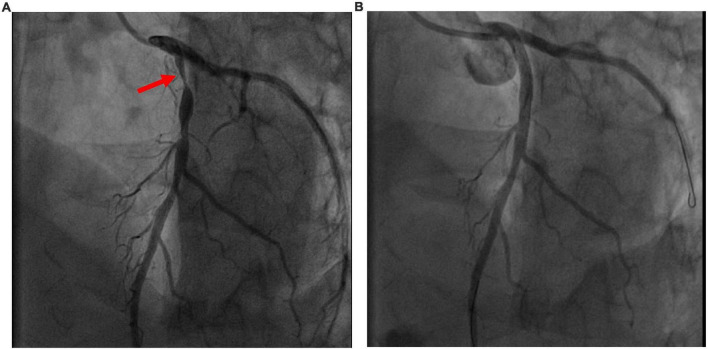
**(A)** Pre- intervention image. Coronary angiogram showed severe proximal left anterior descending artery stenosis (red arrowhead). **(B)** Post- intervention image. Post- stenting coronary angiogram.

**FIGURE 2:**
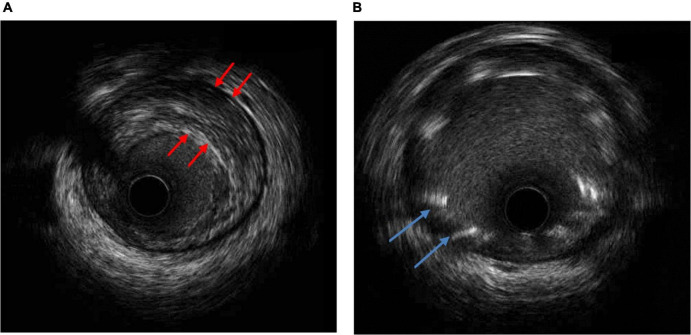
**(A)** Intravascular ultrasound (IVUS) of the narrowest lesion site showing a fibrotic plaque (red arrowhead). **(B)** IVUS post-stenting with fair stent expansion without stent edge dissection or stent malapposition (blue arrowhead).

A previously healthy 35-year-old male, non-smoker, who had a cardiopulmonary arrest 2 min after receiving the Oxford- AstraZeneca (ChAdOx1 nCoV-19) COVID-19 vaccination was referred to our hospital. The patient was a physical education teacher at a high school with a normal body mass index of 22.5 kg/m^2^. No prior chest pain or syncope events were ever experienced. At the vaccination center, he collapsed 2 min after being injected, and cardiopulmonary resuscitation (CPR) was performed immediately. The automated external defibrillator showed shockable rhythm, and he was defibrillated four times before return of spontaneous circulation. The total duration of CPR was 15 min. Intubation was done at a local medical hospital 30 min later, and he was then transferred to our hospital for further management after 3 h. Upon arrival, he was unconscious, with a Glasgow Coma Scale score of E1V1M3, and intermittent general tonic clonic seizure activity was noted. Anaphylaxis was highly suspected at first, but no significant skin-mucosal tissue involvement was noticed on physical examination. There were no signs of respiratory compromise or gastrointestinal symptoms at the vaccination center. A tryptase test was done to rule out anaphylaxis, and it was within normal limits. He had an elevated troponin I level of 1.04 ng/ml (reference range <0.3) approximately 4 h after cardiopulmonary arrest, however, electrocardiography did not show ST-elevation. Transthoracic echocardiography showed a left ventricular ejection fraction of 73% without regional wall motion abnormalities. He was then admitted to the intensive care unit for targeted temperature management. Followed up troponin I levels increased to 5.226 ng/mL 6 h later but decreased to 2.231 ng/mL the next day. He had normal cholesterol, low- density lipoprotein, triglyceride and glycohemoglobin levels. No events of life-threatening arrhythmias were recorded in the intensive care unit. After 3 days, he regained consciousness and was extubated 1 day later. A coronary angiogram was performed, which showed severe stenosis at the proximal left anterior descending artery (Figure 1A). The stenosis was pre-dilated with a semi-compliant 2.5 mm balloon, and then intravascular ultrasound was used to determine the plaque composition, as well as the length and size of the stent. A fibrotic plaque was identified with a length of around 35 mm (Figure 2A). The distal lumen of the artery was 4.0 mm with a plaque burden of <50%. A 4.0 mm non-compliant balloon was used for further pre-dilatation of the stenosis, followed by stenting with a 4.0 mm × 38 mm drug eluting stent (Figure 1B). Final post-dilatation of the stent was done with the 4.0 mm non-compliant balloon. Post-stenting intravascular ultrasound showed an adequate landing zone and stent expansion without stent edge dissection or stent malapposition (Figure 2B). He was discharged 2 days later without significant complications. At a follow-up visit 3 months after discharge he was asymptomatic, and he remained on dual-antiplatelet therapy.

## Discussion

To the best of our knowledge, no similar case of sudden cardiorespiratory collapse immediately after COVID-19 vaccination has been reported. According to the Council for International Organizations of Medical Sciences (CIOMS) and the World Health Organization (WHO) Working Group on Vaccine Pharmacovigilance, an adverse event following immunization (AEFI) is defined as any untoward medical occurrence following immunization which does not necessarily have a causal relationship to the vaccine. The adverse event may be any unfavorable or unintended sign, abnormal laboratory finding, symptom or disease. CIOMS and WHO have defined five cause-specific AEFI, including (i) reaction to a vaccine product, (ii) reaction to a defect in vaccine quality, (iii) reaction to an immunization error, (iv) reaction related to anxiety, and (v) coincidental event, to differentiate vaccine- and vaccination-related reactions from coincidental events by assignment of causality (4). Most reported AEFIs are not serious, however, uncommon adverse reactions such as anaphylaxis, myocarditis and thrombosis have been reported after administering a vaccine (3). In the phase 3 trial of the Oxford-AstraZeneca (ChAdOx1 nCoV-19) COVID-19 vaccine, the incidence rates of adverse events were similar in the vaccinated and placebo groups, with the most common being general pain, headache, and injection-site pain. The incidence rates of serious and medically attended adverse events such as potential immune-mediated conditions were reported to be low and similar to the placebo group. In addition, no anaphylaxis events were reported (5). However, real world studies of Oxford-AstraZeneca (ChAdOx1 nCoV-19) COVID-19 vaccine have however reported severe hypersensitivity reactions with major cardiovascular parameter changes (6).

Another common adverse effect shortly after many types of vaccination among adolescents and young adults is syncope (vasovagal or vasodepressor), which may complicate non-allergic systemic reactions. A vasovagal event is more likely to be triggered by anxiety and pain, causing a drop in heart rate, and blood pressure, rather than the COVID-19 vaccine itself. The Centers for Disease Control and Prevention in the United States reported the rate of post-immunization syncope in 2006 among people >5 years of age was 0.054/100 000 doses distributed and about 80% of syncope events usually self-resolve and occur within 15 min after vaccination (7). In 2018, WHO proposed a new term “immunization stress-related response (ISRR)” to cover the entire spectrum of manifestations (symptoms and signs) of a stress response before, during or immediately after immunization. ISRR may demonstrate as acute stress responses, vasovagal reactions or dissociative neurological symptom reactions with or without non-epileptic seizures. Early presentations of an ISRR may be an acute stress response due to sympathetic involvement with increased heart rate and blood pressure and symptoms may vary from mild feelings of worry to difficulty in breathing. Occasionally, this may be followed by an over-compensatory parasympathetic response in which heart rate and/or blood pressure fall sharply resulting in a vasovagal reaction characterized by symptoms ranging from dizziness to syncope (8). In this patient, it is difficult to distinguish if the syncopal event or a new-onset ventricular arrhythmia led to cardiac arrest. However, as no life-threatening arrhythmias were noted throughout hospitalization, it is deemed more likely that the vasovagal reaction caused a transient ischemia of the myocardium due to a very high-grade chronic stable lesion, initiating tachyarrhythmias leading to cardiac arrest (9). To define the relationship between AEFI and COVID-19 vaccination, the causality WHO algorithm could be adopted to determine the direct link between vaccination and adverse effects as shown in many studies (10, 11). According to the WHO algorithm, our case has consistent causal association to immunization and the vasovagal event is likely to be an immunization anxiety-related reaction.

Other most frequently discussed major cardiovascular (CV) complications after COVID-19 vaccination include myocarditis, pericarditis and vascular thrombotic events, however, rare cases of acute coronary syndrome and cardiac arrest have also been reported (12, 13). In a study of data from the WHO, Kaur et al. reported that the most common CV adverse events observed with the COVID-19 vaccines under study were tachycardia, palpitations and flushing. A case series in the UK on the AstraZeneca vaccine reported 167 cases of cardiac arrest (including 35 fatalities), 386 cases of myocardial infarction (MI) (including 51 fatalities), and 79 cases of acute MI (including 13 fatalities) (14). In 2 studies showing CV complications associated with COVID-19 vaccines, the time gap between vaccination and the incidence of MI varied from 15 min to 2 days, with several possible mechanisms proposed such as immune thrombotic thrombocytopenia, a series of allergic reactions leading to occlusion of coronary arteries, and high demand and low supply due to vaccination stress in weaker patients, where else the time gap for myocarditis varied from 24 h to 7 days after receiving Oxford-AstraZeneca (ChAdOx1 nCoV-19) COVID-19 vaccine (15, 16). However, it is still unclear whether there is any link between COVID-19 vaccines and MI, and further longitudinal studies are needed to clarify this issue.

Besides COVID-19 vaccination and its AEFIs, the timing of coronary angiography and percutaneous coronary intervention after successful resuscitation from out-of-hospital cardiac arrest (OHCA) without ST-segment elevation electrocardiography was another issue in our case. Given that he did not have electrocardiographic evidence of myocardial ischemia and as he had a normal left ventricle systolic function without regional wall motion abnormalities on transthoracic echo, coronary angiography could possibly have been foregone. The prevalence of coronary artery disease (CAD) has been reported to be around 60–70% among those successfully resuscitated from OHCA without ST-segment elevation myocardial infarction (STEMI) (17, 18). Although most studies do not support a routine early invasive strategy in OHCA patients without STEMI or signs of refractory cardiogenic shock, performing coronary angiography after neurological recovery to try and identify the cause of the arrest seemed to be reasonable in our case owing to the high incidence of CAD in these studies (17–19).

## Conclusion

With the increasing number of people receiving COVID-19 vaccinations, rare but serious AEFIs have been reported. We hope that this case report will raise awareness among healthcare providers worldwide that in the era of COVID-19 vaccination, even though fatal AEFIs are rare, heightened awareness of severe side effects needing medical attention is very important.

## Data availability statement

The raw data supporting the conclusions of this article will be made available by the authors, without undue reservation.

## Ethics statement

Written informed consent was obtained from the individual(s) for the publication of any potentially identifiable images or data included in this article.

## Author contributions

CC took care of the patient and wrote the report. C-PL and C-JC performed the cardiac angiography and intravascular ultrasound. P-HC revised in the report. All authors contributed to the article and approved the submitted version.
